# Microfluidic co‐culture devices to assess penetration of nanoparticles into cancer cell mass

**DOI:** 10.1002/btm2.10079

**Published:** 2017-09-26

**Authors:** Maria Jarvis, Michael Arnold, Jenna Ott, Kapil Pant, Balabhaskar Prabhakarpandian, Samir Mitragotri

**Affiliations:** ^1^ Biomolecular Sciences and Engineering Program University of California Santa Barbara CA 93106; ^2^ Dept. of Molecular, Cellular and Developmental Biology University of California Santa Barbara CA 93106; ^3^ Dept. of Chemical Engineering University of California, Center for Bioengineering Santa Barbara CA 93106; ^4^ Biomedical Technology, CFDRC Huntsville AL 35806; ^5^ Present address: John A. Paulson School of Engineering and Applied Sciences, Harvard University Cambridge MA 02138

**Keywords:** drug delivery, drug discovery and development, microfludic device, nanocrystals, nanoparticles

## Abstract

In vitro and in vivo assessment of safety and efficacy are the essential first steps in developing nanoparticle‐based therapeutic systems. However, it is often challenging to use the knowledge gained from in vitro studies to predict the outcome of in vivo studies since the complexity of the in vivo environment, including the existence of flow and a multicellular environment, is often lacking in traditional in vitro models. Here, we describe a microfluidic co‐culture model comprising 4T1 breast cancer cells and EA.hy926 endothelial cells under physiological flow conditions and its utilization to assess the penetration of therapeutic nanoparticles from the vascular compartment into a cancerous cell mass. Camptothecin nanocrystals (∼310 nm in length), surface‐functionalized with PEG or folic acid, were used as a test nanocarrier. Camptothecin nanocrystals exhibited only superficial penetration into the cancerous cell mass under fluidic conditions, but exhibited cytotoxicity throughout the cancerous cell mass. This likely suggests that superficially penetrated nanocrystals dissolve at the periphery and lead to diffusion of molecular camptothecin deep into the cancerous cell mass. The results indicate the potential of microfluidic co‐culture devices to assess nanoparticle‐cancerous cell interactions, which are otherwise difficult to study using standard in vitro cultures.

## 1 INTRODUCTION

Canonical drug delivery research usually commences with the validation of a carrier or a drug using in vitro static cell cultures in which cells are grown in 2D monolayers and are subjected to the drug and subsequently tested through a variety of established methods for cellular uptake and cytotoxicity effects. If efficacy and toxicity outcomes in the static cultures are deemed satisfactory, then the carriers are advanced to in vivo studies. Currently, on average, five compounds from the initial pool of 5,000–10,000 enter clinical trials, and only one becomes a successful FDA approved drug.^1^ Since carriers often alter the drug's efficacy and toxicity, drug‐carrier combinations must also go through the same rigorous validation and approval process. This approach limits the likelihood and speed of translation of in vitro foundational research to in vivo outcomes.^2^

The knowledge gap between the performance of the carriers in vitro and in vivo is often difficult to bridge due to the disparate nature of the two methods of studies. In vitro cell cultures are typically conducted under static conditions and use a monoculture. In vivo studies, by definition, involve a dynamic environment where a multitude of contributing factors could collectively dictate the outcome and it is often difficult to isolate confounding elements and elucidate the mechanistic differences between in vivo and in vitro observations.

Static 2D monolayer cell cultures do not fully account for the impact of physiologically relevant shear forces on carriers. Physiological flows in blood and interstitium are often laminar—dominated mostly by viscous forces and diffusive mixing within higher micro‐ and macro‐regime vessel sizes.^3^ In addition, standard in vitro cultures lack the multicellular environment containing complex extracellular matrix, which is characteristic of tissues. These physical parameters strongly impact carrier performance in vivo, for example, the carrier's ability to extravasate and accumulate at the cancerous cell mass site.

Microfluidic devices offer the potential to bridge the gap between the standard in vitro and in vivo models for drug delivery and discovery because of their ability to integrate physiological processes which are often overlooked or not directly accounted for in traditional in vitro methods.^4^ In comparison to traditional in vitro models, data from microfluidics devices can provide a more accurate and comprehensive prediction of how well a carrier will perform in vivo.

In this study, we utilized an idealized co‐culture microfluidic device (ICD) with an inner tissue culture chamber and two flanking outer vascular channels connected to the tissue chamber via micron sized pores.^5^ The inner tissue culture chamber of the ICD was cultured with murine breast cancer cell line, 4T1, and the outer vascular channels were cultured with the human umbilical vein endothelial cell line, Eahy.926. Cancerous and healthy cells were cultured in 3D in the tissue chamber and exposed to camptothecin (CPT) nanocrystals, with rod‐shaped morphology, under physiologically relevant shear stresses found within micro‐domain sized vessels.^6^ Cells subjected to nontoxic and nonimmune reactive nanoparticles under physiologically relevant shear stresses are known to induce cell death due to the physical and mechanical interactions of particles and cell surfaces which are enhanced and impart a cytotoxic effect.^7^ The devices were used to monitor penetration and efficacy of the nanocrystals within the cancerous cell mass site after short infusion time periods, akin to bolus injections.

The choice of therapeutics (camptothecin nanocrystal) was motivated by our previous studies, which demonstrated the benefits of rod‐shaped nanoparticles over spheres.^8^ Nanocrystals provide a unique ability to increase drug loading as well as control its release kinetics.^9^ The crystalline nanorods used here comprise entirely of camptothecin, a Topo I inhibitor. Hydrophobic drugs have traditionally posed a challenge in drug delivery due to their poor solubility and dependence on amphiphilic carriers for their distribution.^10^ Nanocrystals posit an alternative to the traditional hydrophobic drug carriers since they are entirely comprised of the hydrophobic drug; creating a high concentration of drug in a localized area.^11^, ^12^, ^13^ Camptothecin nanocrystals were used either in their bare form or surface‐modified to display PEG or PEG‐folic acid. Folic acid was chosen for its ability to target the folic acid receptor on 4T1 cells.^14^, ^15^, ^16^

## 2 METHODS

### 2.1 Preparation of camptothecin nanocrystals

All CPT nanocrystals were prepared using the solvent diffusion method. Unmodified camptothecin nanocrystals (CPT‐UM) were used as a base model. To prepare PEG‐modified camptothecin nanocrystals (CPT‐PEG), DSPE PEG2K amine was added concurrently during the formation of the CPT nanocrystals. Folic acid‐modified camptothecin nanocrystals (CPT‐FA) were prepared by first conjugating DSPE PEG2K amine to folic acid and then adding it during CPT nanocrystal preparation.

To make CPT nanocrystals, 5 ml of 0.8 mg/ml of CPT (Sigma Aldrich) in DMSO solution was pipetted dropwise into a 120 ml water mixture containing 1% w/w alpha—tocopherol (Sigma). The mixture was stirred at 800 rpm under constant ultrasonication at room temperature (22°C) for 1 hr. CPT‐UM nanocrystals formed at the boundary where DMSO diffused into the water. The CPT‐UM nanocrystals were then centrifuged three times at 20°C with milliQ water (18.2 Ω) at 3,500 rpm. The concentration of CPT‐UM nanocrystals was determined by dissolving the nanocrystals in DMSO and reading the absorbance at 366 nm using a spectrophotometer (Tecan M220 Infinite Pro) and a CPT calibration curve.

To prepare CPT‐PEG nanocrystals, a mixture of 5 ml of 0.8 mg/ml CPT and 3.2 mg/ml DSPE PEG2K Amine (Avanti Polar Lipids) was added to the 1% alpha—tocopherol water mixture solution, all subsequent steps for preparation, purification, and quantification described above for CPT‐UM nanocrystal preparation were followed. The successful incorporation of DSPE PEG2K amine was validated via X‐ray diffraction (XRD) of DSPE PEG2K amine, CPT, and alpha‐tocopherol in their free powder form compared to the CPT‐PEG construct (Panalytical Empyrean Powder Diffractometer). CPT was quantified using absorbance at 366 nm as described above. DSPE PEG2K amine was quantified via Phosphorous solution state NMR (P^31^) (Varian 600 MGHz). Spectra were analyzed using Mnova software; peaks were integrated and compared to a K_2_HPO_4_ standard to determine quantities of phosphorous present. Phosphorous and DSPE PEG2K Amine are present in 1:1 molar ratios enabling us to quantify the milligrams of DSPE PEG2K amine present from phosphorous signals.

To prepare CPT‐FA, DSPE PEG2K amine‐FA conjugates were prepared first. Specifically, 4.5 mg of folic acid was dissolved in 500 µl of DMSO. This solution was then added to 100 µl of 5 mg/ml EDC (Sigma) in DMSO solution. It was then vortexed and rotated for 30 min at room temperature. To this solution, 19 mg of DSPE PEG2K amine dissolved in 500 µl of DMSO was added; this combined mixture was vortexed and rotated overnight at room temperature. The DSPE PEG2K amine—folic acid conjugate was then purified with a HyperSep C18 octadecyl uncapped bonded silica column, with an acetonitrile—milliQ H_2_O (18.2 Ω) 5–50% v/v gradient. Polymer‐folic acid conjugate eluents were then analyzed via Matrix Assisted Laser Desorption Ionization‐Mass Spectrometery (MALDI‐MS, Microflex LRF A Bruker) and with FTIR set to 24 scans and taken in acetonitrile (Magna IR 850 Nicolet). FTIR spectra were analyzed in the fingerprint region using OMNIC software.

To incorporate folic acid into CPT crystals, 5 ml of 0.8 mg/ml of CPT (Sigma Aldrich) in DMSO solution was pipetted dropwise into a 120 ml water mixture containing 1% w/w alpha—tocopherol (Sigma). The 20% acetonitrile—milliQ H_2_O (18.2 Ω) eluent containing PEG‐FA conjugate was then added dropwise to this solution and the overall mixture containing CPT, DSPE PEG2K amine, and FA was stirred at 800 rpm with constant ultrasonification at room temperature (22°C) for 1 hr. After 1 hr, the CPT‐FA nanocrystals were then centrifuged three times at 20°C with milliQ water (18.2 Ω) at 3,500 rpm. The presence of folic acid was quantified using absorbance at 290 and 370 nm, and CPT was quantified using fluorescence at 366/434 nm—both utilized a spectrophotometer (Tecan M220 Infinite Pro) and a CPT and FA calibration curve at all respective wavelengths. DSPE PEG2K Amine was quantified using P^31^ NMR as described above in CPT‐PEG constructs.

Morphologies of CPT, CPT‐PEG, and CPT‐FA nanocrystals were analyzed using a scanning electron microscope (SEM). Surface charges of all nanocrystalline scaffolds suspended in 1x PBS pH 7.4 were measured as zeta potential using a Nanoseries‐Zetasizer (Malvern).

### 2.2 CPT release from CPT‐UM and CPT‐PEG nanocrystals

Drug release of CPT from CPT‐UM and CPT‐PEG was achieved using Slide‐A‐Lyzer MINIdialysis Devices of 3.5k MWCO (Life Technologies, Grand Island, NY). Freshly prepared nanocrystals were resuspended in 500 μl of citric acid buffered solutions pH 3 and 5, a PBS buffered solution of pH 7.4, RPMI cell culture media containing 10% FBS and 1% Pen–Strep, and lastly, 100% FBS in dialysis cups. Dialysis devices were inserted into microcentrifuge tubes containing 1 ml of the corresponding solution placed at 37°C and set on an orbital shaker at 100 rpm. At indicated time points, aliquots of CPT were removed from the microcentrifuge tubes and CPT concentration as determined via absorbance. Corresponding solutions were then added at each time point to maintain a constant volume.

### 2.3 Cell culture

All cell lines were commercially obtained from ATCC and were grown in a humidified incubator with 5% CO_2_ at 37°C. Endothelial cell line, EA.hy926 cells, were cultured using DMEM medium supplemented with 10% FBS and 1% penicillin–streptomycin (Pen–Strep). Murine mammary tissue cancerous cell line, 4T1 cells, were cultured using RPMI‐1640 medium supplemented with 10% FBS and 1% Pen–Strep.

### 2.4 Idealized co‐culture microfluidic devices nanocrystalline penetration studies

**Figure 1:**
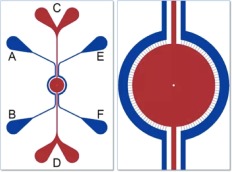
Cartoon depictions of the idealized co‐culture microfluidic devices (SynVivo), channels in blue represent the outer vascular while red channels represent the inner tissue culture channel used in this study for breast cancer cell culture. A zoomed in image of the central tissue culture chamber better depicts the slits connecting the outer and inner tissue culture channels

Idealized co‐culture microfluidic devices (ICD's) were purchased from SynVivo (Cat#102016). See Figure 1. Dimensions were set to the following: an outer channel (OC) of 200 microns, a travel distance (*T*) of 50 microns, slit spacing (*S*_s_) of 50 microns, and a slit width (*W*_s_) of 2 microns.

ICD's outer vascular channels were coated with 100 µg/ml human fibronectin (Thermo Fisher), subjected to 5 PSI N_2_ (laboratory grade) for 15 min, and incubated in a humidified incubator with 5% CO_2_ at 37°C in preparation for seeding with EA.hy926 cells. A schematic of the ICD's is provided below. Once cultured, EA.hy926 cells were allowed to incubate for 4 hr to attach to the outer vascular channels of the ICDs before changing the media using a syringe pump set at 4 µl/min (KD Scientific Inc.). Inner tissue culture chambers of ICDs were subsequently coated with a 20% v/v Matrigel (Corning) cell culture medium slurry. To facilitate polymerization of the Matrigel, ICDs were incubated with 5% CO_2_ at 37°C for 1 hr before infusion of freshly prepared CPT‐UM nanocrystals. For the duration of the experiments, Eahy.926 cells received media changes every 12 hr at 2 µl/min for 10 min using a syringe pump (KD Scientific Inc.). Freshly prepared CPT‐UM nanocrystals were then infused through the outer vascular channels at 1 mg/ml, 4 µl/min, for 15 min and imaged at 48 hr post infusion using an inverted microscope (Olympus CKX‐41). Near‐UV and FITC filters were used to obtain images of CPT‐UM nanocrystal penetration and cell autofluorescence respectively.

### 2.5 In vitro cytotoxicity of CPT‐UM, CPT‐PEG, and CPT‐FA

In vitro activity of CPT‐UM, CPT‐PEG, and CPT‐FA nanocrystals in 4T1 cells was analyzed using Calcein AM and Ethidium homodimer‐1 of the Live‐Dead assay kit (Invitrogen). 4T1 cells were cultured in 96‐well plates at a density of 50,000 cells in 100 μl of RPMI, 10% FBS, 1% Pen–Strep. Cells were allowed to attach overnight before undergoing exposure of nanocrystals for short incubation times. 4T1 cells in short incubation experiments were treated with nanocrystals for 3 hr then washed with RPMI, 10% FBS, 1% Pen–Strep and allowed to incubate for 48 hr before being assayed. All nanocrystal treatments were subjected to a serial dilution series starting with 1 mg/ml of CPT as determined by absorbance and fluorescence methods described above. The following controls were used in 4T1 cells: free CPT (Sigma), Folic Acid (Fisher), and DSPE PEG2K amine (Avanti Polar Lipids Inc.). At 48 hr, 4T1 live cells were measured using the Live‐Dead Assay Kit (Invitrogen) and analyzed using a plate reader (Tecan M220). For quantification of the number of live cells, 1 μM of Calcein AM was added to the cells and incubated for 30 min before taking fluorescence intensity readings (ex./em. 495/530 nm). Fluorescence backgrounds were subtracted from each reading. Assays were performed in quadruplicate in three independent experiments. The results are expressed in IC_50_ format determined from dose response curves generated for each nanocrystalline scaffold via the Chou‐Talalay method.

### 2.6 Idealized co‐culture microfluidic devices

The inner and tissue culture chamber and outer vascular channels of the ICDs were prepared as described above for EaA.hy.926 cell seeding. Unlike the ICDs used for nanocrystal penetration studies, the inner tissue culture chambers of the ICDs were cultured with 4T1 cells 24 hr post EAa.hy.926 seeding. 4T1 cells were cultured in a 20% v/v Matrigel (Corning) cell culture medium slurry. 4T1 cells were allowed 4 hr to adhere to the outer inner tissue culture chambers of the ICDs before conducting a 100% cell culture media change using a syringe pump set at 4 µl/min (KD Scientific Inc). EA.ahy.926 and 4T1 cell cultured ICDs were then maintained in a humidified incubator with 5% CO_2_ at 37°C. Every 12 hr Eahy.926 and 4T1 cells received 100% media changes with their respective media at 2 µl/min until all experiments were terminated.

## 3 RESULTS

### 3.1 Synthesis and characterization of camptothecin nanocrystals

**Figure 2:**
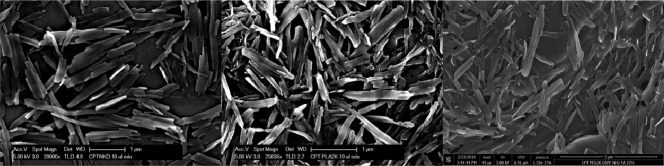
Scanning electron microscopy showing the morphology and size of CPT‐UM, CPT‐PEG, and CPT‐FA

Rod‐shaped CPT nanocrystals were prepared using the solvent diffusion method and were visualized using SEM (Figure 2).

The dimensions of the nanocrystalline rods are reported in Table 1; the average long and short axes of these particles are 270 nm, 67 nm for the CPT‐UM, 348 nm, 54 nm for the CPT‐PEG, to 395 nm, 81 nm for the CPT‐FA. In the case of CPT‐PEG and CPT‐FA, the w/w ratio of CPT/DSPE PEG2K amine and CPT/FA are 0.25 and 0.88, respectively. The percent yield of PEG retained on the surface of the CPT nanocrystal was approximately 84%. CPT‐UM, CPT‐PEG, and CPT‐FA nanocrystals all possess negative zeta potentials, although the CPT‐PEG and the CPT‐FA constructs are significantly more positive compared to CPT‐UM (Table 1). SEM images of the constructs depict aggregation which is attributed to the drying and coating method of the nanocrystals on the SEM mounts. Overall the morphology of particles was not altered by surface modification, which was expected since the surface coating is expected to be thin.

**Table 1:** Z—Size averages with polydispersity indices, the Zeta potential, and the width as determined by ImageJ from SEM images are reported here

Sample	Z—size average (nm)	Zeta potential (mV)	Width (nm)
CPT‐UM	271.7 PDI: 0.174	−30.4 ± 6.61	67 ± 17
CPT‐PEG	348.0 PDI: 0.155	−9.66 ± 4.94	54 ± 9
CPT‐FA	395.2 PDI: 0.172	−4.67 ± 3.57	81 ± 21

**Figure 3:**
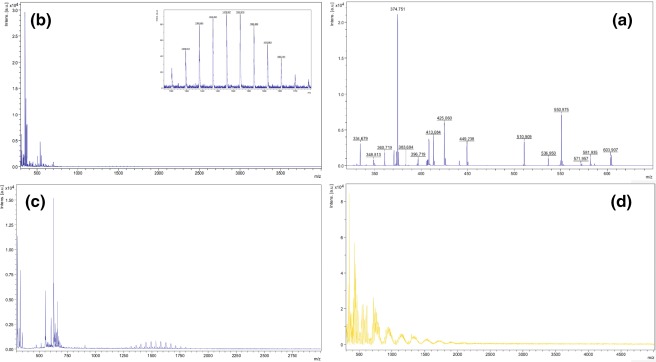
Matrix assisted laser desorption ionization‐mass spectroscopy (MALDI‐MS) spectra of (a) CPT UM (b) CPT‐PEG (c) CPT‐FA, and (d) DSPE PEG2K AMINE‐FA conjugate

Mass spectra of the CPT‐UM construct showed the parent peak at 348 m/z. CPT‐PEG spectra depicted the polymeric PEG signature centered around 1,500 m/z. Peaks labeled in the zoomed inlet are 44 m/z apart from one another corresponding to the weight of one PEG unit. CPT‐FA spectra also show a polymeric signature centered around 1,500 m/z. Lastly, mass spectra of the DSPE PEG2K amine‐FA conjugate has polymeric signatures ranging from 800 to 1,700 m/z (Figure 3).

**Figure 4:**
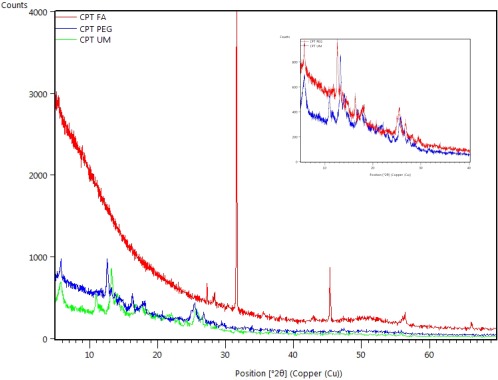
X‐ray diffraction spectroscopy of (Green) CPT‐FA (Blue) CPT‐PEG and (Red) CPT‐UM crystals. Inlet depicts zoom‐in of the CPT‐PEG and CPT UM spectral patterns

XRD spectra of all constructs verified the nanocrystalline nature of the particle scaffolds. See Figure 4. The distinct signatures of CPT‐UM, CPT‐PEG, and CPT‐FA signified that the three constructs are nanocrystalline in nature, and they possess unique packing structures. Most significantly, it is apparent that CPT‐PEG is not a CPT‐UM nanocrystal encased in a liposome but has the lipid tail of DSPE PEG2K amine either anchored within the hydrophobic regimes of CPT‐UM crystal, or the DSPE PEG2K Amine has physically adhered to the nanocrystalline surface in such a way it significantly alters the crystalline pattern and creates a distinction between CPT‐UM and CPT‐PEG. This distinction carries over and is further enhanced by the conjugation of folic acid to DSPE PEG2K Amine resulting in distinct nanocrystalline patterns for all three constructs.

DSPE PEG2K amine‐FA conjugates were analyzed using Fourier transform infrared spectroscopy (FTIR). Mass spectra of dissolved CPT‐FA nanocrystals were taken in conjunction with FTIR spectra and absorbance measurements for folic acid at 290 nm were used to verify and quantify the presence of the folic acid in the construct.

**Figure 5:**
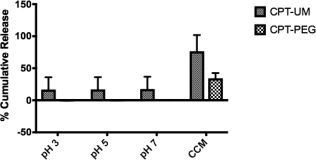
Cumulative release (%) of Camptothecin (CPT) measured at 366 nm in pH 7.4, pH 5, pH 3, and DMEM containing 10% FBS and 1% Pen–Strep (Cell Culture Medium) at 37°C under constant orbital shaking for CPT‐UM crystals and CPT‐PEG crystals. Percent cumulative release calculated as percentage of release compared to 100% release of CPT‐UM crystals in 100% FBS under the same physical conditions

The release of free CPT from all three nanocrystals was measured. CPT‐PEG was stable and did not break down releasing free CPT during the 5‐day extended analysis in buffered solutions of pH 7, pH 5, and pH 3. Release studies conducted in cell culture media (DMEM, 10% FBS, 1% penicillin–streptomycin) revealed CPT‐PEG broke down and released 34% of the CPT encased in the construct. These values represent a significant decrease in the release of free CPT for the CPT‐PEG construct compared to the release from CPT‐UM. The release of CPT from CPT‐UM construct was observed in buffered solutions pH 7, pH 5, and pH 3 but to a far lesser extent than in cell culture media for the same construct. The above results are consistent with the slight solubility, approximately 3 µg/ml, of CPT in buffered pH 3 solution.^17^, ^18^ Hundred percent release of CPT was observed when CPT‐UM particles were placed in 100% FBS and allowed to incubate for 5 days under constant orbital shaking. All release data for CPT‐UM and CPT‐PEG were normalized to this positive control. The delayed effect of CPT release was attributed to the surface coating (Figure 5).

### 3.2 In vitro cell growth inhibition by CPT nanocrystal constructs

The effects of CPT‐UM, CPT‐PEG, and CPT‐ FA on in vitro growth inhibition of 4T1 cells were assessed. The effects of folic acid and free CPT were also tested for in vitro growth inhibition of 4T1 cells. PEG and its fatty acid derivatives are nonimmunogenic and biologically inert.^19^ Folic acid by itself exhibited minimal toxicity regardless of exposure time. Short exposure time experiments which, are more applicable toward the framework of microfluidic device experiments had IC_50_ values of 26 µg/ml, 650 µg/ml, and 560 µg/ml, respectively for the CPT‐UM, CPT‐PEG, and CPT‐FA constructs (Table 2).

**Table 2:** 4T1 cell line IC_50_ values reported here for CPT‐UM, CPT‐PEG, and CPT‐FA for short exposures of *t* − 3 hr with end time points set at 48 hr. IC_50_ values were determined using Chou‐Talalay methods describing Log(*F_a_*/*F_u_*)= *m*Log(*D_x_*)*–* Log(*D_m_*), IC_50_ values taken from *D_m_* calculations

Sample	IC50 value (µg/ml)
CPT‐UM	26
CPT‐PEG	650
CPT‐FA	560

### 3.3 Cell growth inhibition in idealized co‐culture microfluidic devices

**Figure 6:**
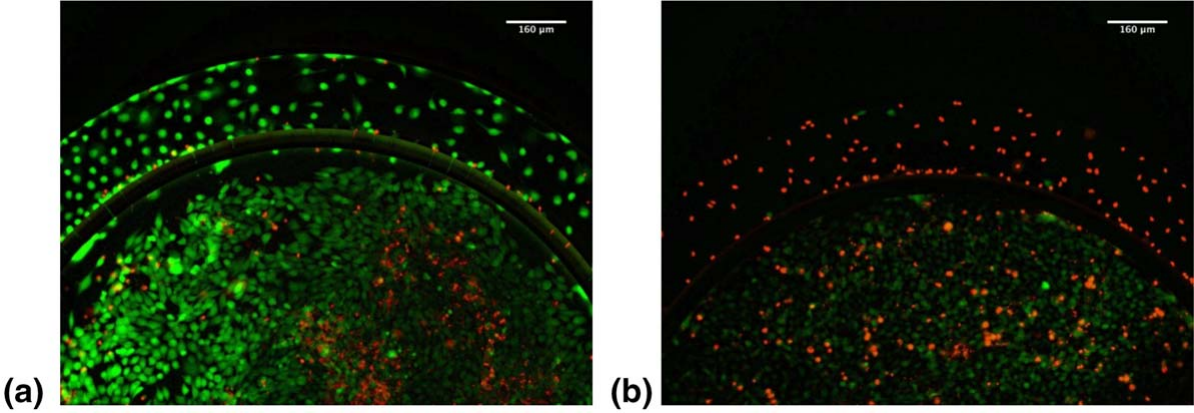
Idealized co‐culture devices cultured with Eahy.926 and 4T1 imaged at 10x with cell death/live fluorescent indicator dyes. Live cell/Green Channel and Dead cell/Red Channel depicted at *t* = 48 hr: (a) control ICD and a (b) test ICD

The effect of all CPT constructs on in vitro growth inhibition of 4T1 cells was assessed within idealized co‐culture microfluidic devices. Significant effect of CPT‐UM on cell viability was observed (Figure 6). In control devices (not exposed to CPT‐UM), a large population of live cells (green) was seen in endothelial chambers as well as within the cancer cell chamber (Figure 6a). A certain fraction of dead cells was seen as well, especially within the cancer cell chamber. This could be attributed to moderate cell death during the seeding process which can compromise cell membrane integrity as the cells are transported through the syringe needle at relatively fast flow rates in a top down fashion resulting in relatively high impact forces at the moment of seeding. A significantly elevated population of dead cells was observed in CPT‐UM‐treated ICDs (Figure 6b). The effect was clear in the endothelial chamber as well as within the cancer cell chamber. Qualitative and quantitative analyses of fluorescent images taken of test ICDs subjected to CPT‐UM nanocrystal flow reveal a significant difference in dead/live cell fluorescence intensity ratios measured from cell viability assays of 4T1 cells within the inner tissue culture chamber.

**Figure 7:**
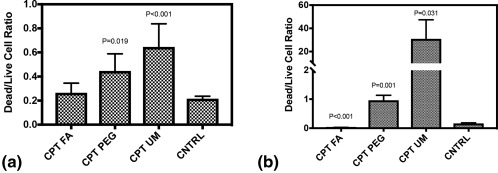
Effect on cell death in (a) 4T1 cells, cancerous cell mass channel, whereby CPT FA and (b) Eahy.926 cells, endothelial channel. Cells were cultured in *n* = 3 idealized co‐culture microfluidic devices. CPT‐UM, CPT‐PEG, and CPT‐FA were flowed at 4 μl/min to a total volume of 500 μl. Devices were incubated for 48 hr before being stained with cell death/live indicator dyes or stains

Effect of CPT nanocrystals on cells was quantified as the difference in the intensity ratio of dead/live cells between CPT‐treated and control cells (nontreated) (Figure 7). CPT‐UM yielded the most prominent effect on cell viability, both in the endothelial as well as cancer cell chamber. CPT‐UM yielded about threefold enhancement in dead/live ratio compared to controls. The effect in the vascular channel was significantly more prominent compared to the cancer cell chamber. For example, CPT‐UM exhibited over 100‐fold increase in dead/live ratio for endothelial cells. This clearly indicates that CPT nanoparticles are significantly more effective in killing endothelial cells compared to cancerous cell mass cells. CPT‐PEG also yielded a significant effect on cell viability in cancerous cell mass and vascular channels. Conversely, a relatively small effect was seen for CPT‐FA in either channel (Figure 7). The effect of CPT is distributed throughout the cancerous cell mass and cytotoxicity is observed even at the middle of the cancer cell chamber. No strong spatial dependence of anti‐cancerous cell mass activity of CPT nanocrystals was found (Figure 7).

### 3.4 Nanocrystal penetration within idealized co‐culture microfluidic devices

**Figure 8:**
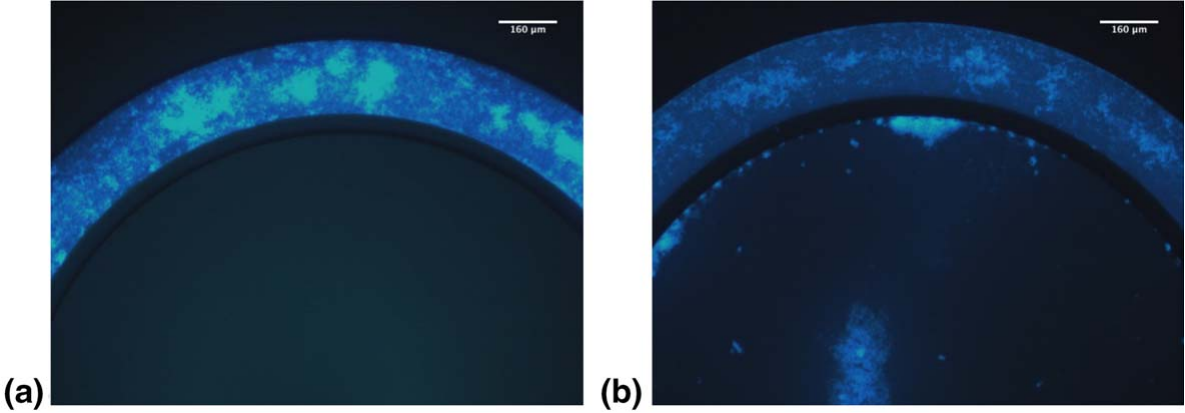
A co‐culture idealized microfluidic device coated with a fibronectin basement membrane was imaged immediately after flow (*t* = 0 hr) with CPT‐UM crystals under the Near UV Channel at 4x and 10x as depicted from left to right to track the progress of the CPT‐UM UV fluorescent nanocrystals through the outer channel into the inner tissue culture chamber. Fluorescent images obtained on an Olympus CKX‐41. (a) ICD *with* endothelial cells. (b) ICD *without* endothelial cells

**Figure 9:**
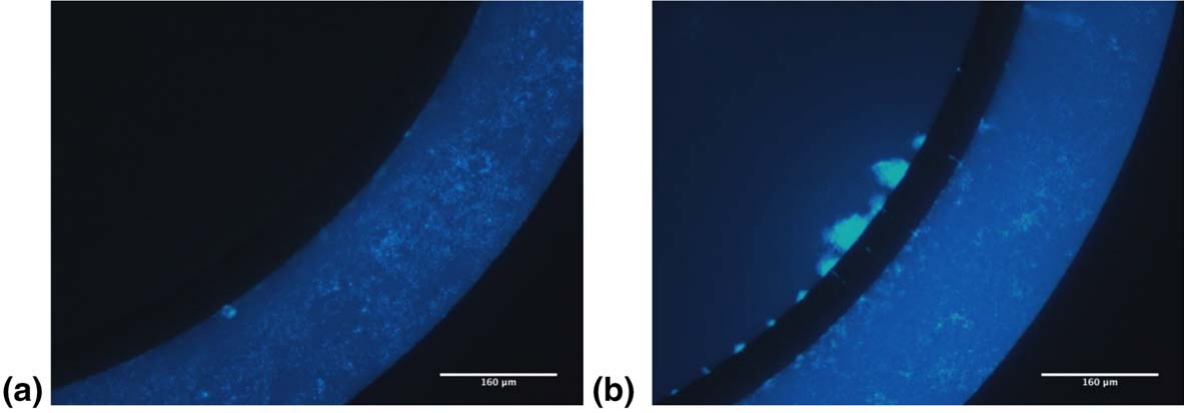
A co‐culture idealized microfluidic device coated with a fibronectin basement membrane was imaged immediately after flow (*t* = 0 hr) with CPT‐UM crystals under the Near UV Channel at 20x as depicted from left to right to track the progress of the CPT‐UM UV fluorescent nanocrystals through the outer channel into the inner tissue culture chamber. Fluorescent images obtained on an Olympus CKX‐41. (a) ICD *with* endothelial cells. (b) ICD *without* endothelial cells

To assess the correlation between the cytotoxicity of CPT nanocrystals and their cancerous cell mass penetration, we assessed migration of CPT‐UM in ICDs with an intact endothelial barrier in the vascular channel. CPT‐UM nanocrystals were localized largely in the vascular channel and no clear penetration was observed into the cancer cell chamber (Figure 8a). To assess whether the limited penetration originated from the endothelial layer, the same experiments were performed using ICDs without endothelial cells. Clear penetration of CPT‐UM was seen in the ICDs in the absence of endothelial cells (Figure 8b). Migration of CPT‐UM could be clearly seen through the slits into the inner chamber. Penetration of CPT even deep within the inner chamber could be seen. Zoomed views of the endothelial‐cancerous cell mass interface further clarify this observation (Figure 9a,b). In the absence of endothelial cells CPT nanocrystals could be seen to penetrate through the slits and appear within the cancer cell chamber (Figure 9b). In the presence of endothelial cells, very limited penetration of CPT‐UM could be seen in the slits and no visible penetration could be seen within the cancer cell chamber (Figure 9a). These results clearly show that endothelial cells pose a barrier for nanoparticle transport into the cancerous cell mass.

## 4 DISCUSSION

Nanoparticles developed for therapeutic applications typically advance through the canonical channels of drug delivery research, whereby the core platform is developed in vitro and then shunted into in vivo research routes. This serial assessment is not ideal and limits the likelihood of successful outcomes.^1^, ^2^, ^3^, ^20^, ^21^, ^22^, ^23^, ^24^, ^25^, ^26^ This study aims to couple conventional in vitro assays with microfluidics to assess nanoparticle penetration, diffusion, and eventual killing of a localized cancerous cell mass while being subjected to physiologically relevant shear stresses, approximately 1 dyne/cm^2^, in a three‐dimensional co‐culture cellular environment. Shear stresses were calculated using the Haagen–Poisseuille equation.

Three nanocrystal platforms were chosen CPT‐UM, CPT‐PEG, and CPT‐FA for studies. While the purpose of the PEG‐coating on the nanocrystals was to extend their half‐life in vivo,^19^, ^27^, ^28^, ^29^ we sought to assess whether the PEG coating would impact dissolution and penetration. Conjugation to folic acid was aimed to improve targeting to folic acid receptors which are known to be upregulated or expressed in breast cancer lines, 4T1 cells included.^14^, ^16^, ^30^

All three nanocrystalline constructs were imaged under SEM to verify their nanorod morphology. As the complexity of the scaffold increased so did their size and charge. Their overall charge remained negative, which has significant implications in their toxicity, uptake, and penetration both in vitro and within the ICDs.^31^, ^32^, ^33^, ^34^, ^35^

Studies described here demonstrate the use of microfluidic co‐culture devices to assess efficacy of nanoparticles and potentially other therapeutics while subjected to flow. The primary distinctive result obtained with microfluidic devices is the lack of nanoparticle penetration deep into the cancerous cell mass. Whereas the nanoparticles readily traversed into the center of the device in the absence of the endothelial layer, minimal penetration was seen in the presence of endothelial cells. These studies verify the presence of a barrier created by the endothelial cells for nanoparticle penetration (Supporting Information Figures 4 and 5). Nevertheless, a clear effect of CPT on survival of cancer cells was observed. In fact, the cytotoxic effect of CPT was seen through the cancerous cell mass, even at the center of the cancerous cell mass. These results suggest that CPT nanocrystals likely dissolve near the periphery of the cancerous cell mass and diffusion of molecular CPT is responsible for cytotoxic effect. This is consistent with the observation that LC50 of CPT nanocrystals correlated with their dissolution rates. CPT‐UM constructs exhibited the highest therapeutic efficacy (Figure 8). The in vitro cell viability data correlated well with the ICD cell viability data. CPT‐PEG and CPT‐FA exhibited lesser efficacy than CPT‐UM likely due to these constructs' slower dissolution (Figure 5). The presence of the PEG‐Folate construct in CPT‐FA nanocrystals was able to reduce nonspecific death observed within the vascular channels (Supporting Information Figure 3).

The data presented here confirm the potential of CPT nanocrystalline scaffolds for cancerous cell mass treatment. The nanocrystals were delivered under a bolus injection‐like infusion method. While minimal‐to‐none penetration of CPT nanocrystals was found in the cancerous cell mass, sufficient amount appeared to cross the endothelium to treat cancer cells. The limited ability of nanocrystals to penetrate into the cancerous cell mass did not appear to originate from the challenge of margination/wall contact. Specifically, in the absence of endothelial cells, nanocrystals readily penetrated into the cancer cell chamber. The primary resistance appeared to arise from the endothelial cell barrier. The ICD's ability to provide insight into carrier behavior under these complex conditions cannot be tested in static in vitro cell culture. The overarching combined results from the in vitro cell culture and the ICD data lead to the conclusion that modest amounts of nanocrystals diffuse into the periphery of the cancerous cell mass site which dissolve and supply drug deep into the cancerous cell mass.^9^ It is the diffusion of small molecule, which results in cell death throughout the entirety of the cancer cell chamber.

Dissolution of nanocrystals appears to play a key role in its efficacy since the nanocrystals themselves were unable to penetrate deep within the cancerous cell mass. CPT‐UM nanocrystals had the highest release rates of CPT. The release profile data suggested that CPT's increased solubility arose from its interactions with serum proteins.^36^, ^37^ Nanocrystalline constructs containing PEG or PEG‐FA coating exhibited increased IC50 values compared to CPT‐UM. Folate receptors (FR's) are known to be overexpressed in a variety of breast cancer cell lines.^16^ For 4T1 cells, FR's are also slightly overexpressed on their cell surfaces.^14^ Some decrease in IC50 value was seen for CPT‐FA compared to CPT‐PEG, thus suggesting the role of FA targeting. However, the effect was modest, thus indicating that the primary effect of CPT appears to be through drug dissolution.

## 5 CONCLUSION

Nanocrystals provide an attractive platform for delivery of highly hydrophobic drugs. In addition to validating and rigorously analyzing CPT nanocrystalline constructs for their properties and performance in vitro, this study aimed to provide an additional cellular analytical tool to help bridge the gap between in vitro characterization and development and in vivo testing. Cell viability data obtained using the idealized co‐culture microfluidic devices cultured with both 4T1 breast cancer and Ea.hy926 endothelial cells in a three‐dimensional construct, in close cellular contact and with flow and shear stress factors included, was insightful and supports that notion of superficial yet impactful delivery of nanocrystals. The CPT nanocrystals' capacity to induce significant levels of cell death within the cancerous cell mass supports further development for in vivo assessment.

## Supporting information

Additional Supporting Information may be found online in the supporting information tab for this article.

Supporting Figures
