# Supplementary material for: Microfluidic co‐culture devices to assess penetration of nanoparticles into cancer cell mass
Source: Bioeng Transl Med. 2017 Sep 26;2(3):268–77. doi: 10.1002/btm2.10079 (PMC5689499; doi:10.1002/btm2.10079)
Supplement: Supplementary file 1 — Supporting Figures [file BTM2-2-268-s001.docx]

**Supporting Data**


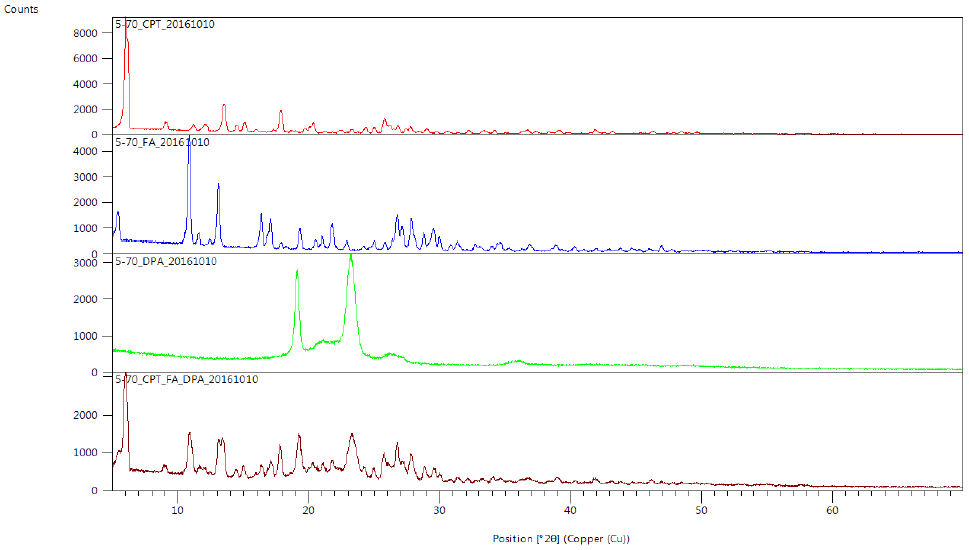


Figure 1A: X-Ray Diffraction Spectra were obtained using a Panalytical MRD Pixel 3D between 5-70 2θ angles. (A) free CPT, folic acid, and DSPE PEG2K Amine (DPA) were analyzed separately and then together in a 1:1:1 mixture of the three free powders as distributed from the manufacturer in their powder form. Plots were generated using HighScore Plus software for XRD control analyses of the nanocrystalline construct CPT-FA.


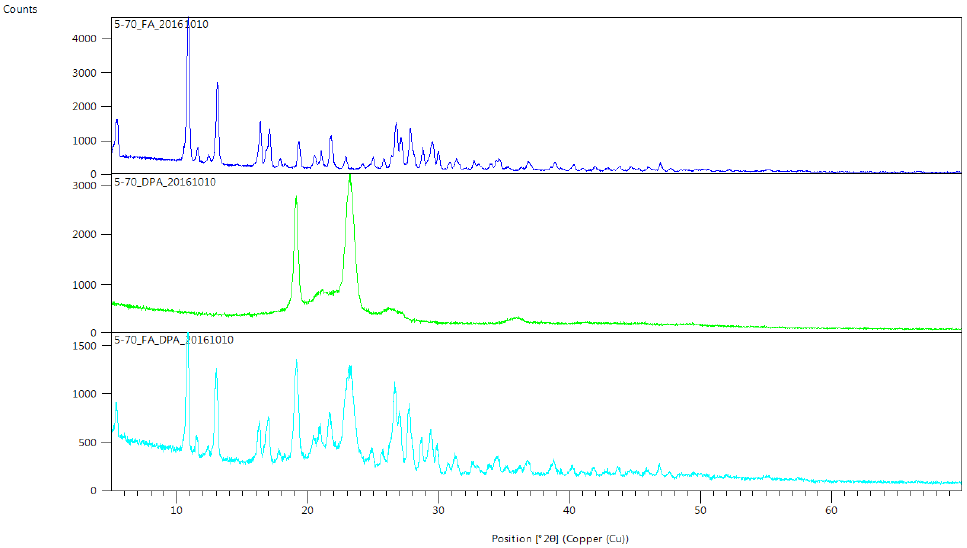


Figure 1B: X-Ray Diffraction Spectra were obtained using a Panalytical MRD Pixel 3D between 5-70 2θ angles. (B) free folic acid and DSPE PEG2K Amine (DPA) were analyzed separately and then together in a 1:1 mixture of the two free powders as distributed from the manufacturer in their powder form. Plots were generated using HighScore Plus software for XRD control analyses of the DSPE PEG2K Amine-FA construct.


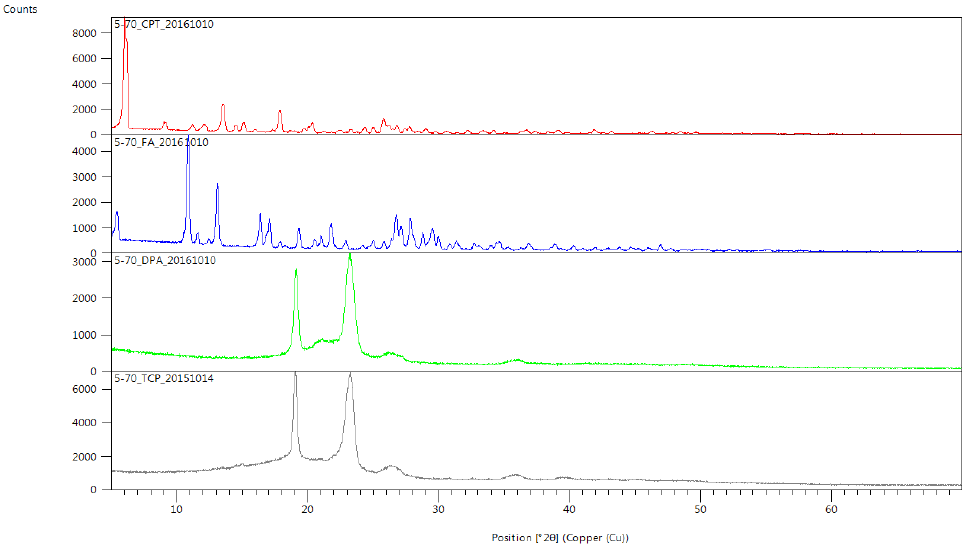


Figure 1C: X-Ray Diffraction Spectra were obtained using a Panalytical MRD Pixel 3D between 5-70 2θ angles. (C) free CPT, folic acid, DSPE PEG2K Amine (PEG), and α-tocopherol (TCP) were analyzed separately as free powders distributed from the manufacturer in their solid form. Plots were generated using HighScore Plus software for XRD control analyses of the nanocrystalline constructs CPT UM, CPT-PEG, and CPT-FA.


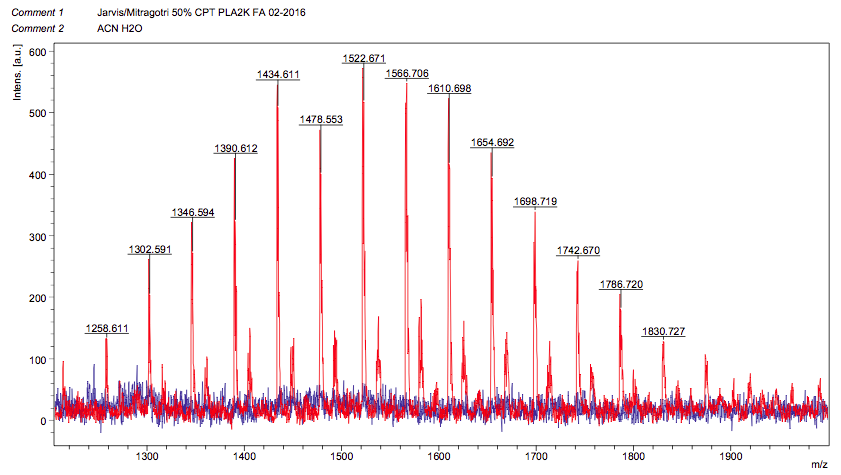

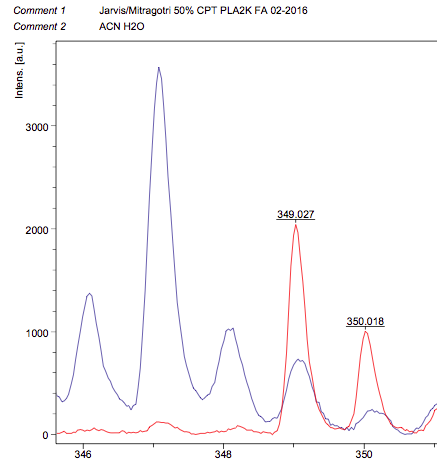


Figure 2: Matrix Assisted Laser Desorption Ionization – Mass Spectrometery (MALDI-MS) spectra were obtained using a Microflex LRF A Bruker (Bruker Daltonics) Microflex LRF MALDI TOF with a 60 Hz nitrogen laser. Background matrix of 2,5-Dihydroxybenzoic Acid was analyzed using FlexImaging software, the spectra is indicated in blue. CPT-FA nanocrystals were digested in acetonitrile, incubated with matrix – spectra is indicated in red. The zoomed inlay shows the camptothecin parent peak, the primary spectra depicts the conjugated PEG polymeric signature.

1. (B)

Figure 3: (A) CPT PEG and (B) CPT FA uptake were measured using fluorescence microscopy, fluorescent images were captured on an Olympus CKX-41 and quantified using ImageJ. N=16 Regions of Interest (ROI’s) were imaged within each well, and studies were conducted N=3 times.


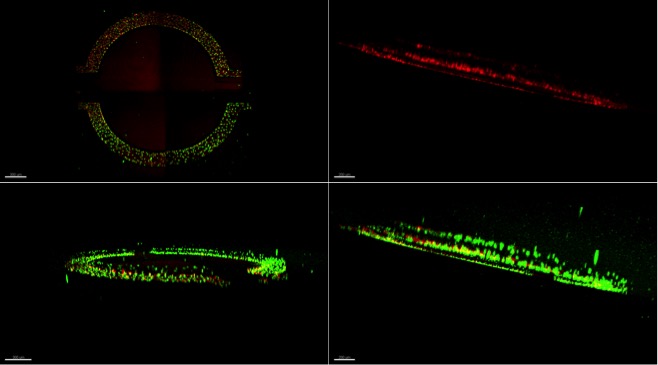


Figure 4: Test ICD stained with NucBlue (Green) and primary and secondary antibodies for ZO-1 (Red). Imaged using the multiarea time lapse feature on the Olympus Fluoview 1000 Spectral Confocal, stiched together using Fluoview software and stacked to create 3D rendering using Imaris imaging software.
